# Proteome of normal human perilymph and perilymph from people with disabling vertigo

**DOI:** 10.1371/journal.pone.0218292

**Published:** 2019-06-11

**Authors:** Hsiao-Chun Lin, Yin Ren, Andrew C. Lysaght, Shyan-Yuan Kao, Konstantina M. Stankovic

**Affiliations:** 1 Department of Otolaryngology, Harvard Medical School, Boston, Massachusetts, United States of America; 2 Eaton Peabody Laboratories and Department of Otolaryngology, Massachusetts Eye and Ear, Boston, Massachusetts, United States of America; 3 Program in Speech and Hearing Bioscience and Technology, Harvard Medical School, Boston, United States of America; 4 Harvard Program in Therapeutic Science, Harvard University, Boston, United States of America; University of Connecticut Health Center, UNITED STATES

## Abstract

The vast majority of hearing loss, the most common sensory impairment, and vertigo, which commonly causes falls, both reflect underlying dysfunction of inner ear cells. Perilymph sampling can thus provide molecular cues to hearing and balance disorders. While such “liquid biopsy” of the inner ear is not yet in routine clinical practice, previous studies have uncovered alterations in perilymph in patients with certain types of hearing loss. However, the proteome of perilymph from patients with intact hearing has been unknown. Furthermore, no complete characterization of perilymph from patients with vestibular dysfunction has been reported. Here, using liquid-chromatography with tandem mass spectrometry, we analyzed samples of normal perilymph collected from three patients with skull base meningiomas and intact hearing. We identified 228 proteins that were common across the samples, establishing a greatly expanded proteome of the previously inferred normal human perilymph. Further comparison to perilymph obtained from three patients with vestibular dysfunction with drop attacks due to Meniere’s disease showed 38 proteins with significantly differential abundance. The abundance of four protein candidates with previously unknown roles in inner ear biology was validated in murine cochleae by immunohistochemistry and in situ hybridization: AACT, HGFAC, EFEMP1, and TGFBI. Together, these results motivate future work in characterizing the normal human perilymph and identifying biomarkers of inner ear disease.

## Introduction

**Fig 1:**
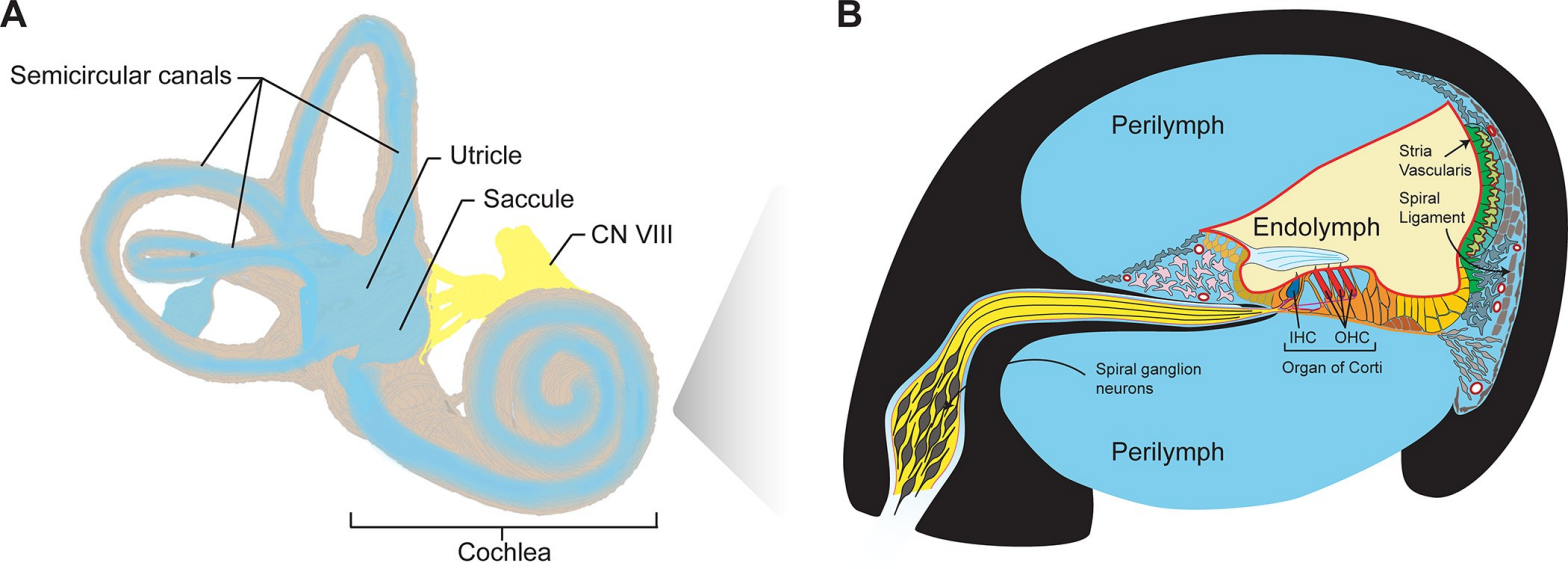
Schematic of the inner ear and cochlear cross section. **(A)** The inner ear includes the organ of hearing (i.e. the cochlea), and five vestibular end-organs: the saccule, utricle and three semicircular canals. Modified after Blausen.com staff (2014). "Medical gallery of Blausen Medical 2014". WikiJournal of Medicine 1 (2). DOI:10.15347/wjm/2014.010. ISSN 2002-4436. **(B)** Perilymph (blue) is the proximal fluid of the inner ear that bathes most cells in the cochlea and fills the scala vestibuli and scala tympani. IHC, inner hair cells (dark blue) and OHC, outer hair cells (red) are part of the organ of Corti. Endolymph fluid is pseudocolored in light yellow. Other structures of interest are labelled as shown, and include the stria vascularis (green), fibrocytes of the spiral ligament (gray), fibrocytes of the spiral limbus (pink), spiral ganglion neurons (SGNs, bright yellow), different types of supporting cell of the epithelial gap junctional network (different shades of orange).

Hearing loss is the most common sensory impairment in humans and it currently disables 466 million people across the globe; this number is expected to rise to 900 million by 2050 [1]. Nearly two-thirds of the population aged over 70 in the United states is affected by disabling hearing loss [2]. A vast majority of this burden is due to sensorineural hearing loss (SNHL), which originates from defects in the cochlea, the spiraling organ of the inner ear (**Fig 1**). In addition, a third of the general population in the U.S. report vestibular symptoms such as vertigo, a persistent spinning sensation [3,4]. The majority of vertigo originates from the balance organs within the inner ear (**Fig 1**). When both the hearing and balance parts of the inner ear are affected, this can lead to audiovestibular pathologies such as in Meniere’s disease (MD), which is characterized by fluctuating hearing loss, vertigo, tinnitus and aural fullness. When vertiginous attacks become incapacitating and hearing loss turns profound, surgical removal of the inner ear’s vestibular organs (*via* labyrinthectomy) provides an effective treatment for vertigo when conservative medical therapy fails. In addition, labyrinthectomy also provides a rare opportunity to access the inner ear tissue in living humans.

The human inner ear is a small, three-dimensionally complex, fluid-filled structure encased in the otic capsule, the densest bone in the body, and located deep in the base of skull. For these reasons, routine clinical biopsy of the inner ear is currently not possible to establish a cellular-level diagnosis, inform disease prognosis or guide targeted therapies. The contemporary assessment of the severity of SNHL and vestibular dysfunction (VD) relies on a combination of semi-quantitative indirect measurements, including pure-tone audiometry (PTA), word recognition (WR) testing, auditory brainstem response (ABR) recordings, and vestibular battery testing. Conventional imaging modalities such as computed tomography (CT) and magnetic resonance imaging (MRI) have limited spatial resolution (0.5 to 1 mm, respectively). Therefore, they detect only significant malformations of major inner ear structures but fail to provide microstructural or cellular details. Although *post-mortem* studies of human temporal bone histopathology have offered invaluable insights into cellular correlates of SNHL and peripheral VD [5], these studies do not offer information in real-time to aid in clinical decision making and management of disease. Therefore, an ideal clinical diagnostic test should rely on a biomarker that 1) allows assessment of the extent of cellular damage in the inner ear and 2) can be sampled and analyzed to provide information in real-time. Such a biomarker would serve as a platform for development and validation of novel, targeted therapies that emerge from ongoing inner ear regenerative research.

There is growing interest in developing a “liquid biopsy” of the inner ear as a surrogate for tissue biopsy to identify molecular biomarkers. Liquid biopsy is based on the sampling of perilymph–the extracellular fluid that bathes most cell types in the inner ear and is enriched in proteins that these cells secrete. Within the cochlea, perilymph percolates the scala tympani and scala vestibuli (**Fig 1**). Previously, the human perilymph has been shown to be of diagnostic value for various etiologies of SNHL [6,7]. Total protein concentration is significantly elevated in the perilymph of patients with vestibular schwannomas (VS), an intracranial tumor that arises from the vestibular nerve and causes SNHL in 95% of patients [6,8]. The first quantitative assessment of the human perilymph proteome was described nearly thirty years ago, where proteins including albumin, transferrin and immunoglobulins were discovered as major components [9]. This was subsequently refined by Thalmann *et al*. where disease-specific protein patterns in patients with perilymphatic fistulas were identified using two-dimensional gel electrophoresis [10]. These reports led to the separation of over 100 proteins and subsequent identification and quantitation of approximately 30 proteins in the perilymph fluid. The first complete human perilymph proteome was assembled by Lysaght *et a*l. using liquid chromatography with tandem MS (LC-MS/MS), where 271 unique proteins were identified across four samples from patients with VSs or undergoing cochlear implantation (CI), and 71 proteins were found to be common to all [11]. Elsewhere, Schmitt *et al*. utilized intraoperative sampling of perilymph from patients with SNHL undergoing CI or VS resection, and identified over 200 proteins unique to the human perilymph that are not present in reference cerebrospinal fluid (CSF) or plasma. Furthermore, comparative analyses between adult and pediatric patients led to the identification of 32 proteins solely found in the perilymph of children [12]. These studies to date not only highlight the diversity and complexity of the perilymph proteome, but also identify candidate proteins for further validation in both diagnostic and prognostic applications.

In the current study, we used LC-MS/MS to perform the first proteomic analysis of human perilymph in patients with otherwise intact, normal hearing. These samples were obtained from patients with normal audiometric thresholds whose inner ears were sacrificed for access to life-threatening meningiomas of the skull base. Normal perilymph was compared to pathologic perilymph from patients undergoing labyrinthectomy for disabling vertigo with drop attacks due to MD. The spatial expression patterns of four novel proteins identified from the analysis with previously unknown roles in inner ear biology were determined in murine cochlear sections using immunohistochemistry and fluorescence in-situ hybridization (FISH).

## Methods

### Study population, sample collection and preparation

Two perilymph samples from normal hearing patients were collected during clinically indicated transotic and transcochlear resection of petroclival meningiomas. Meningiomas are tumors of the meninges that surround the brain. Because petroclival meningiomas did not directly affect the inner ear, these two patients had clinically normal hearing. One additional sample was obtained from a patient who had a meningioma within the internal auditory canal (IAC) and bilateral symmetric mild SNHL of unknown origin. Three pathologic perilymph samples were obtained during labyrinthectomy from patients with Meniere’s Disease (MD) resulting in ipsilateral profound hearing loss and disabling vertigo with drop attacks. All patients provided informed consent for perilymph collection. All study protocols were approved by the Human Studies Committee of Massachusetts General Hospital and Massachusetts Eye and Ear Infirmary, and conducted in accordance with the Helsinki Declaration.

For all patients, approximately 1 μL of perilymph was collected with a 28-gauge needle inserted through the round window into the scala tympani prior to surgical opening of the cochlea. Each sample was flushed from the needle using 200 μL of sterile phosphate buffered saline (PBS) and immediately stored at -80°C.

### Gel electrophoresis and LC-MS/MS

A total of 70 μL of each diluted specimen was loaded into each of two wells of a 1.0 mm x 5 well Novex 4–20% Tris-Glycine gel (Invitrogen, EC6024BOX). Gels were run and each sample lane was cut into 9 equal-sized sections by molecular weight and the corresponding sections from each of the two lanes were grouped together for further processing. Gel electrophoresis was done for estimation of the sample concentration. Gel was cut to prevent clogging of the tubing used for LC-MS/MS. A tenth section was taken from each gel, from a lane which had been loaded purely with running buffer, to serve as a background control for each gel. Sections were placed in a sterile container and immediately stored at -80°C without any preservative, and transported to the Harvard Faculty of Arts and Sciences Mass Spectrometry and Proteomics Resource Laboratory Core Facility on dry ice. Specimens were processed for LC-MS/MS per established protocols. Briefly, excised gel bands were trypsin digested and analyzed using microcapillary reversed-phase high-performance liquid chromatography (HPLC), coupled via a nano electrospray flex ion source to a linear trap quadrupole (LTQ)-Orbitrap tandem mass spectrometer (ThermoFisher Scientific). Fragments were stored as centroid m/z value and intensity pairs. Sequencing of peptides were performed using the SEQUEST algorithm based on the Uniprot Knowledge base human reference proteome database, and matched to proteins using Proteomics Browser Suite (ThermoFisher).

### Peptide identification, and protein alignment

After processing, peptide identification, and protein alignment of the six samples was completed, the peptide lists were combined and remapped so that identical peptides were always attributed to the same parent protein, regardless of parent sample. During this remapping, the protein that a given peptide had been assigned to most often in the individual analyses was selected to be the global parent. The protein counts for each sample were adjusted to reflect this globally uniform assignment. This procedure was necessary to account for the fact that minor changes in peptide assignment could have highly significant impacts on inferred differential expression.

### Differential expression analyses

We utilized three statistical approaches to infer differential expression between perilymph groups. The results from each approach were then collated to ensure the largest breadth and highest confidence in our differential expression determinations. The three methods were selected because each is a commonly accepted technique to study differential expression from count data; however, each method produces unique results.

Approaches 1 and 2 were performed with the differential expression analysis package *msmsTests*, implemented within *R* and available through Bioconductor [13]. This package was developed to infer differential expression between biological conditions using spectral counts from label-free LC-MS/MS experiments. It can employ several different techniques to model the data, and due to the over-dispersed nature of LC-MS/MS datasets we employed the quasi-likelihood GLM regression model and negative-binomial distribution model derived from *edgeR* [14] (*edgeR* is a differential expression package commonly used for the analysis of RNA-Seq derived count data).

Approach 3 employed *DESeq2* [15], available through Bioconductor and built upon the negative-binomial distribution. This package is commonly applied to RNA-Seq count data but is applicable to MS/MS data. However, while *DESeq2* and *msmsTests-edgeR* employ the same distribution assumption, the processes used to normalize the data, calculate dispersions, and infer differential expression are different between the two methodologies and thus have significant impacts on the end results. Specifically, *DESeq2* employs *size factor*-based normalization rather than total spectral counts as in *msmsTests*. This approach utilizes a geometric mean and weakens the effect that very high count proteins have on the normalization process. Furthermore, *DESeq2* also shares dispersion information across similar expression level proteins to moderate estimates and potentially provide more reliable estimates. Principal component analyses (PCA) was performed on the count matrix normalized by *DESeq2*’s *size-factor* procedure and *regularized* log-transformed.

The *msmsTests-QL* and *msmsTests-edgeR* procedures were conducted per the standard differential expression workflow. Counts were normalized based on the total spectra in each sample. The null model was constructed with a parameter for sample blocking and the alternative model had terms for blocking and tumor hearing group. A post-test filter was applied to remove proteins with an absolute log-fold-change ≤ 1 and with mean spectral counts ≤ 5 in at least one condition. These terms and cutoffs were selected to ensure that statistical inferences were only being made when sufficient evidence was available in the dataset to warrant them. Significance values were corrected for multiple hypotheses using the Benjamini-Hochberg procedure and considered significant for *p* < 0.05.

*DESeq2* was also implemented according to the standard workflow [15]. The design matrix had terms for blocking and tumor hearing group. Significance values were corrected using the Benjamini-Hochberg procedure and considered significant for *p* < 0.05. *DESeq2*’s native independent filtering procedure was utilized to maximize the number of differentially expressed proteins at this alpha value. All three analyses were repeated identically as above on the count data from the control section of each protein gel. This analysis was performed to ensure all differentially expressed proteins were the result of sample material, not gel processing or contamination.

### Ingenuity pathway analysis

For all genes that were significantly differentially expressed (*p* < 0.05) between patients with normal hearing and those with severe vestibular dysfunction, a differential analysis was performed using Ingenuity Pathway Analysis (Qiagen, Redwood City, CA, USA) per previously established protocols [16]. Within IPA, a *pathway* refers to cell signaling and metabolic pathways that have been previously characterized based on existing studies, whereas a *network* refers to regulatory relationships among predefined molecules. A central node within a network is defined as the most interconnected molecule. Statistical analysis was performed as part of IPA using right-tailed Fisher’s exact test. The *p*-value is reported as the likelihood that the association between input genes and a given network or pathway is due to chance. Networks with *p* ≤ 10^−12^ were considered significant.

### Paraffin-embedded cochleae for immunohistochemistry and *in situ* hybridization

Six-week-old C57BL/6 mice were intracardially perfused with 4% paraformaldehyde fixative in 0.1M PBS buffer. Cochleae were extracted and intracochlearly perfused with the same fixative, then post-fixed for two hours and immersed in 10% EDTA for three days on a shaker at room temperature. Decalcified cochleae were dehydrated through a series of increasing ethanol concentrations, transferred sequentially to 50%, 70%, 95%, 100% ethanol baths for one hour each. After removing ethanol by Histo-Clear twice for one hour, samples were infiltrated by molten paraffin wax in the oven for one hour, and then embedded into paraffin wax blocks. Paraffin-embedded cochleae were sectioned at 8 μm thickness for subsequent immunohistochemistry and *in situ* hybridization.

For immunohistochemistry, after antigen retrieval by 1% sodium dodecyl sulfate (SDS) in PBS for 10 min, slides were blocked in 10% horse serum in PBS for one hour. Rabbit anti-alpha-1-antichymotrypsin (LS Bio, WA, USA) and goat anti-myosin Vlla (Proteus Biosciences, CA, USA) in 0.1% Triton-PBS (PBST) were applied to samples overnight at room temperature. Negative control samples were processed simultaneously in the same manner, with the exception that PBST was used to replace primary antibody. Slides were incubated with anti-rabbit Alexa Fluor 568-conjugated and anti-goat Alexa Fluor 488-conjugated secondary IgG (Invitrogen, CA, USA) for one hour. Hoechst dye was placed over each slice twice for 10 min each for labeling nuclear DNA.

For *in situ* hybridization, the digoxigenin (DIG) RNA Labeling Mix (Roche, Basel, Switzerland) and T7 RNA polymerase were used to synthesize the DIG-labeled single-stranded sense and antisense RNA probes of target genes: hepatocyte growth factor activator (*Hgfac*), EGF-containing fibulin-like extracellular matrix protein 1 (*Efemp1*) and transforming growth factor beta induced (*Tgfbi*). The DIG-labeled single stranded sense RNA probes were prepared with T3 RNA polymerase. Slides were de-paraffinized in Histo-Clear and rehydrated in ethanol. Endogenous peroxidase activity was reduced using hydrogen peroxide. Slides were placed in 4% paraformaldehyde (PFA), followed by Proteinase K digestion and treated with triethanolamine and acetic anhydride. The hybridization mix including specific DIG-labeled probes were added to each slide at 50°C overnight. To remove non-specific RNA hybridization, slides were washed with Saline-Sodium citrate. Slides were blocked in Tyramide Signal Amplification reagent (PerkinElmer, USA) for one hour, incubated with anti-DIG POD antibodies for 1.5 hours, treated with the TSA Plus Cyanine 3 System (PerkinElmer, USA) for 15 min to label the targets of interest. All fluorescent images were captured on a microscope equipped with epifluorescent filters and a CCD camera.

## Results

### Principal component analyses of the perilymph proteome

**Fig 2:**
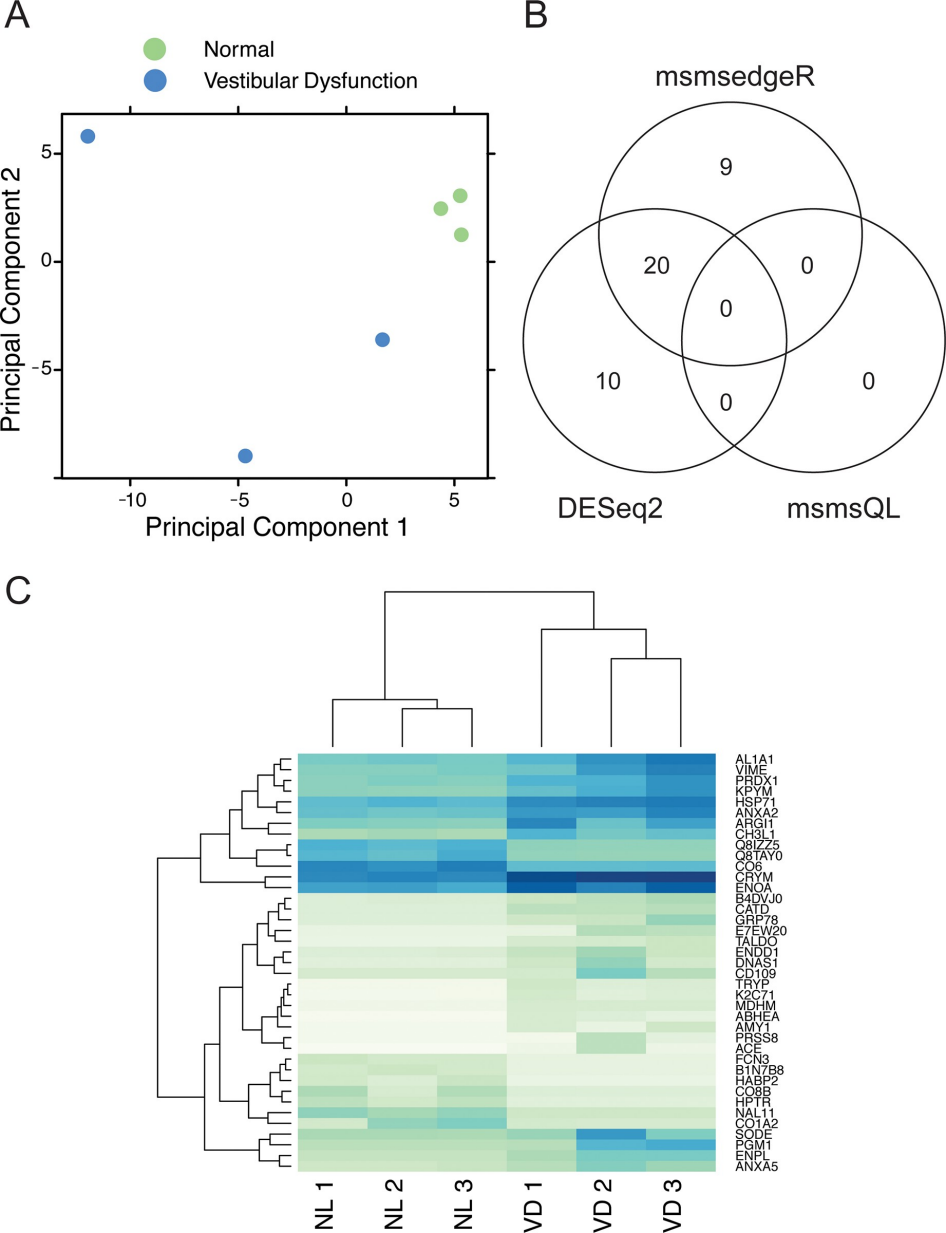
Proteomic analysis of human perilymph. (**A**) Principal component analysis (PCA) of the normalized and log-transformed spectral count matrix from the 6 samples of perilymph, including 3 from patients with normal hearing (pseudocolored green) and 3 from patients with severe vestibular dysfunction (pseudocolored blue). Tight clustering is observed among the clinically normal samples but the vestibular dysfunction samples appear quite heterogeneous. **(B)** Venn diagram of differentially expressed proteins identified by each differential expression analysis approach (DESeq2, msmsedgeR and msmsQL). **(C)** Heat map of proteins differentially expressed between perilymph obtained from patients with clinically normal hearing (NL 1, NL 2, and NL 3) versus those from patients with severe vestibular dysfunction (VD 1, VD 2, and VD 3). Darker blues indicate higher spectral counts. For assembly of the proteome of normal human perilymph, sample NL3 was excluded as it was obtained from a patient with an intracananicular meningioma.

A total of 2,773 proteins were identified from peptide fragments derived from all six clinical samples of human perilymph. Principal component analyses (PCA) revealed that the proteomic profiles of the subset of meningioma patients with normal hearing clearly clustered together and away from the profiles of patients with severe vestibular dysfunction (**Fig 2A**). This pattern of clustering remains true even for the perilymph sample obtained from the patient with an intra-canalicular meningioma and near-normal hearing, which is therefore included as one of the clinically “normal” specimens for subsequent differential expression analysis to improve statistical power. The perilymph proteomes of patients with vestibular dysfunction appeared more heterogeneous and did not cluster along either principal component (**Fig 2A**). This result indicates that subset of patients with normal hearing and vestibular function shares a signature that distinguishes them from other subsets. By contrast, the perilymph proteomic profiles of the subset of patients undergoing labyrinthectomy likely represent distinct molecular pathophysiology that underlie vestibular dysfunction in Meniere’s disease.

### Assembly of the core perilymph proteome

**Fig 3:**
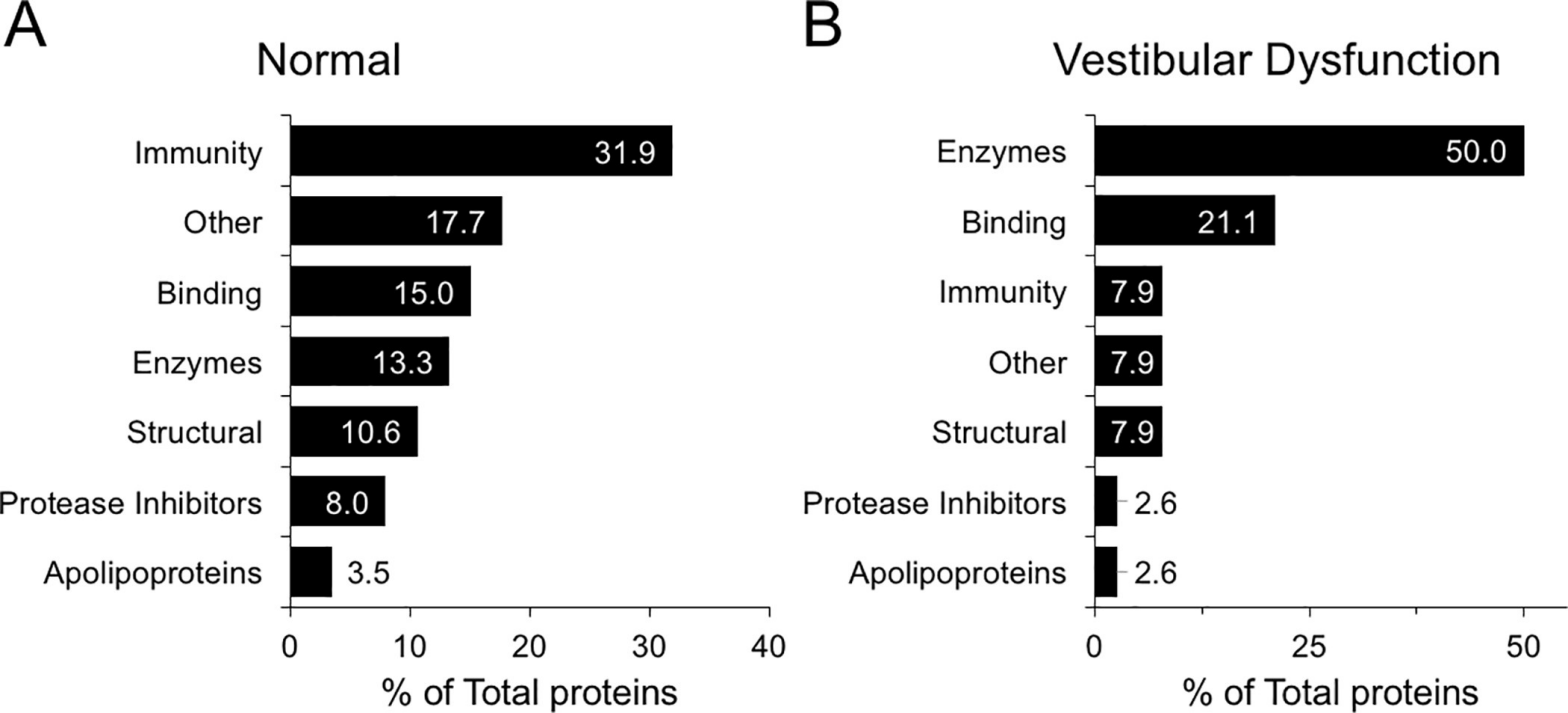
Major protein categories in human perilymph. (**A**) The perilymph of patients with intact hearing. Within each category, the percentage of the total amount of proteins is denoted within each bar. The Other category included proteins involved in ubiquitination, blood coagulation cascade, and receptors. (**B**) Categorization of differentially expressed proteins between perilymph fluid from patients with normal hearing and those with vestibular dysfunction.

To assemble a map of the core proteome of the normal human perilymph, two perilymph samples from patients with meningiomas away from the inner ear and the cochleovestibular nerve were included for analysis. The proteomic profile from the patient with an intra-cananicular meningioma was excluded, as the composition of the perilymph may be altered due to the location of the tumor within the IAC and thereby may confound the analysis. A total of 248 proteins were identified via semi-quantitative analysis of protein abundance based spectral count data, defined as at least 5 spectral counts within both perilymph samples. This analysis generated a list of 228 common proteins of the human perilymph, which represents the most complete mapping of human normal perilymph ever performed to date **(S1 Table)**. This list of 228 proteins was further sub-categorized based on the function of each protein (**Fig 3A**). Proteins within the immunoglobulin and immunoglobulin-related families were present at high concentrations (31.9%), whereas a variety of enzymes such as proteases as well as their respective protease inhibitors comprised 21.3% of the perilymph proteome. This was closely followed by binding proteins (15.0%), structural proteins in the cytoskeleton and extracellular matrix (10.6%), and apolipoproteins (3.5%).

Using Ingenuity Pathway Analysis (IPA), this list of proteins was further mapped to 159 unique genes **(S1 Table)**. IPA also identified highly interconnected networks (**S1 Fig**) as well as significant canonical pathways, which consisted of well-established cytokine signaling and metabolic pathways, such as acute response signaling (*p* = 3.19E-47) and retinoid acid receptor (RXR) activation (*p* = 7.55E-43) (**Table 1**).

**Table 1:** Ingenuity pathway analysis (IPA) of the 228 perilymph proteins from patients with normal hearing. The three top ranking canonical pathways, *p*-values, percent overlap, category definition, and associated function and diseases are shown.

Top canonical pathways	*p* value	Overlap (%)	Category	Top Function and Diseases
1. Acute Phase Response Signaling	3.19E-47	21.6%	Cytokine Signaling	Cellular Movement; Hematological System Development and Function; Immune Cell Trafficking
2. Liver X receptor (LXR) / retinoid X receptors (RXR) Activation	7.55E-43	26.4%	Nuclear Receptor Signaling	Lipid Metabolism; Molecular Transport; Small Molecule Biochemistry
3. Farnesoid X receptor (FXR) / retinoid X receptors (RXR) Activation	1.25E-38	23.8%	Nuclear Receptor Signaling	Lipid Metabolism; Molecular Transport; Small Molecule Biochemistry

Comparing this set with a previously published perilymph proteome of 71 common proteins identified from patients with vestibular schwannoma or undergoing cochlear implantation, there is a significant amount of overlap between the perilymph fluid samples. Our list of 228 proteins included 72% (51 of 71) of those previously identified from pathologic ears **(S2 Table)**, whereas 28% (20 of 71) proteins were unique to the previously published proteome [11]. An additional 90 unique proteins were added to the database **(S2 Table)**. Comparing of our data to a published proteome from the Max-Planck Unified Proteome Database (MAPU) of human cerebral spinal fluid (CSF), there was an overlap of 62% (88 of 141 genes) **(S3 Table)** [17,18]. Comparison of this dataset with proteins from mouse perilymph and CSF revealed less overlap between the two species, with only 11% (15 of 141) genes from the current set present in the list of mouse perilymph proteins **(S4 Table)** [19]. However, this analysis did not consider homology between proteins, only those with identical gene names.

### Differential expression between normal and diseased perilymph

Differential expression between proteins within perilymph of patients with both intact hearing and vestibular function, and those from patients with severe hearing loss and vestibular dysfunction was performed using 3 different methodologies to gain maximal insight. The results are summarized in a Venn diagram (**Fig 2B**). The *DESeq2* approach identified 30 differentially proteins, the *msmsTests-edgeR* approach identified 29 proteins, and the *msmsTests-QL* method did not identify any proteins with differential expression between the two datasets (**S5 Table)**. A total of 20 proteins were found to be shared between *msmsTests-edgeR* and *DESeq2*. Of note, 3 proteins were identified by the *DESeq2* approach as differentially expressed within the three perilymph samples from patients with normal vestibular function. Amongst these, one protein (ARGI1, encoded by *ARG1*), was shared with perilymph from patients with vestibular dysfunction and was therefore excluded from subsequent analyses of differentially expressed proteins.

Hierarchical clustering was performed on the subset of 38 differentially expressed proteins common to all 3 analytical methodologies (**Fig 2C; S5 Table**). Again, proteins from patients with normal vestibular function are more homogeneous and cluster together, which is distinct from the proteomic profiles of patients with Meniere’s Disease (**Table 2; S3 Fig**). Furthermore, certain subsets of proteins were observed to be consistently either up-regulated or down-regulated, when comparing their abundance in the vestibular dysfunction cohort relative to the normal patient cohort.

**Table 2:** Patient demographics. VD1-3, vestibular dysfunction samples 1–3; NL1-3, normal samples 1–3; M/F, male/female; PTA, pure tone average in decibels (dB); WR, word recognition; IT, intratympanic; Ipsi, ipsilateral; Contra, contralateral.

				Ipsi. ear		Contra. ear				
ID	AGE	M/F	R/L	PTA (dB)	WR (%)	PTA (dB)	WR (%)	Pathology	Tumor size (mm)	Duration (yrs)
VD1	63	F	L	56	64	14	100	Intractable Meniere's disease w/ drop attacks, failed IT gentamicin	N/A	4
VD2	76	M	L	69	40	9	92	Intractable Meniere's disease w/ drop attacks, failed IT gentamicin	N/A	> 5
VD3	61	M	R	68	14	4	100	Intractable Meniere's disease w/ drop attacks, failed IT gentamicin	N/A	12
NL1	60	F	R	16	100	18	98	Petroclival atypical meningioma	41 x 29 x 37	N/A
NL2	35	F	R	6	96	2	100	Petroclival atypical meningioma	43 x 41 x 40	N/A
NL3	64	F	R	10	100	8	100	CPA and intracanalicular secretory meningioma	18 x 11 x 29	N/A

To interrogate the molecular signaling pathways represented by the differentially expressed proteins in patients with severe vestibular dysfunction, IPA’s core analysis was performed on our list of 38 proteins. This revealed significant enrichment for signaling pathways involved in inflammatory disease, organismal injury, energy metabolism, developmental disorders, cell-to-cell signaling and interaction, and nervous system development (**Table 3**). This list of 38 proteins was also sub-categorized based on each protein’s function (**Fig 3B**). Amongst the top 3 signaling networks, the differentially expressed proteins extracellular signal regulated kinase (ERK 1/2), heat shock protein (HSP), and amyloid precursor protein (APP) were nodal in each of the networks, respectively (**S2 Fig**).

**Table 3:** Ingenuity pathway analysis (IPA) of the 38 perilymph proteins differentially expressed between patients with normal hearing and those with severe vestibular dysfunction. The three top ranking networks, associated network functions, *p*-values and the nodal molecule with the highest number of connections are shown.

Associated Network Functions	*p* value	Nodal Molecule (Number of connections)
1. Organismal Injury and Abnormalities, Respiratory Disease, Inflammatory Disease	1.0E-48	ERK1/2 (19)
2. Energy Production, Small Molecule Biochemistry, Developmental Disorder	1.0E-13	HSP (18)
3. Cell-To-Cell Signaling and Interaction, Cellular Assembly and Organization, Nervous System Development and Function	1.0E-11	APP (20)

### Expression of AACT protein in the cochlea

**Fig 4:**
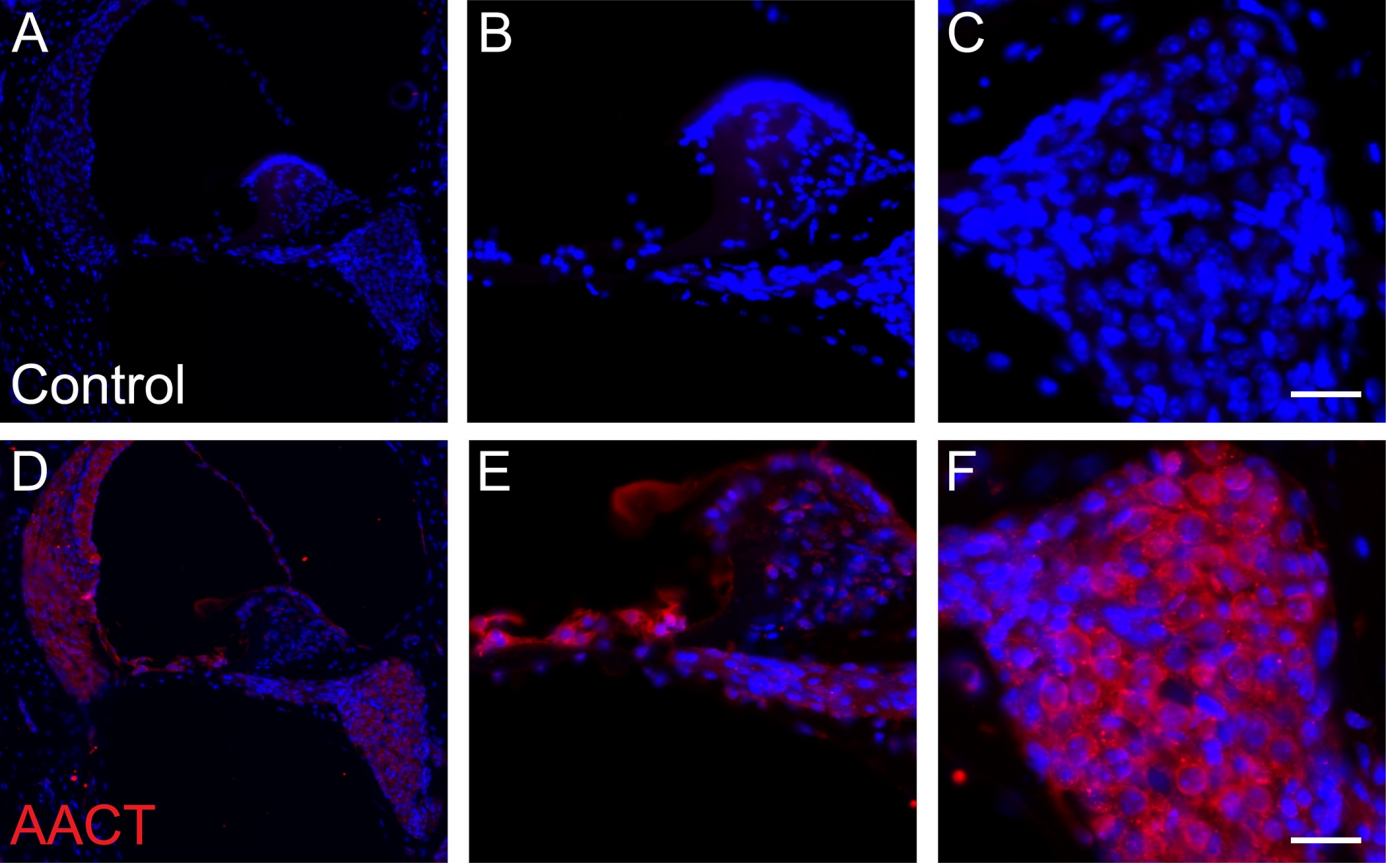
Immunohistochemical validation of AACT protein expression in the murine cochlea. (**A-C**) Negative controls were carried out without primary antibodies for AACT. Panel **B** shows a magnified view of panel **A** focusing on the organ of Corti including inner and outer cell cells. Panel **C** shows a magnified view of spiral ganglion cells. (**D-F**) Immunofluorescent staining of AACT protein (pseudocolored red) demonstrated widespread expression in the cochlea, including spiral ligament and stria vascularis. Panel **E** shows a magnified view of panel **D** focusing on the organ of Corti. Panel **F** demonstrates a magnified view of spiral ganglion cells. Cell nuclei were counterstained with DAPI (pseudocolored blue). Scale bars, 25 μm.

Among 228 proteins constituting the core perilymph proteome, four proteins were selected as candidates for further validation: alpha-1 antichymotrypsin (AACT), hepatocyte growth factor activator (HGFAC), EGF-containing fibulin-like extracellular matrix protein 1 (EFEMP1), and transforming growth factor beta induced (TGFBI). These proteins were selected because *a)* their roles in inner ear physiology are still unknown; *b)* related proteins (i.e. epidermal growth factor [EGF], hepatocyte growth factor [HGF] and transforming growth factor beta [TGFβ]) are known to be crucial for hearing [20–22]; and *c)* mutations in the encoding genes are associated with human disease. Accordingly, immunofluorescence studies were performed to localize these proteins in the inner ear. Only AACT displayed intensive immunoreactivity throughout the cochlear cross sections, including stria vascularis, spiral ligament, the organ of Corti and spiral ganglion neurons (SGNs) (**Fig 4**). In the organ of Corti, substantial immunoreactivity of AACT was observed in inner hair cells (IHCs), outer hair cells (OHCs), the adjoining supporting cells and the tectorial membrane. There was no gradient in AACT expression between the cochlear base and apex.

### *In situ* hybridization of *Hgfac*, *Efemp1*, *Tgfbi* genes in the cochlea

**Fig 5:**
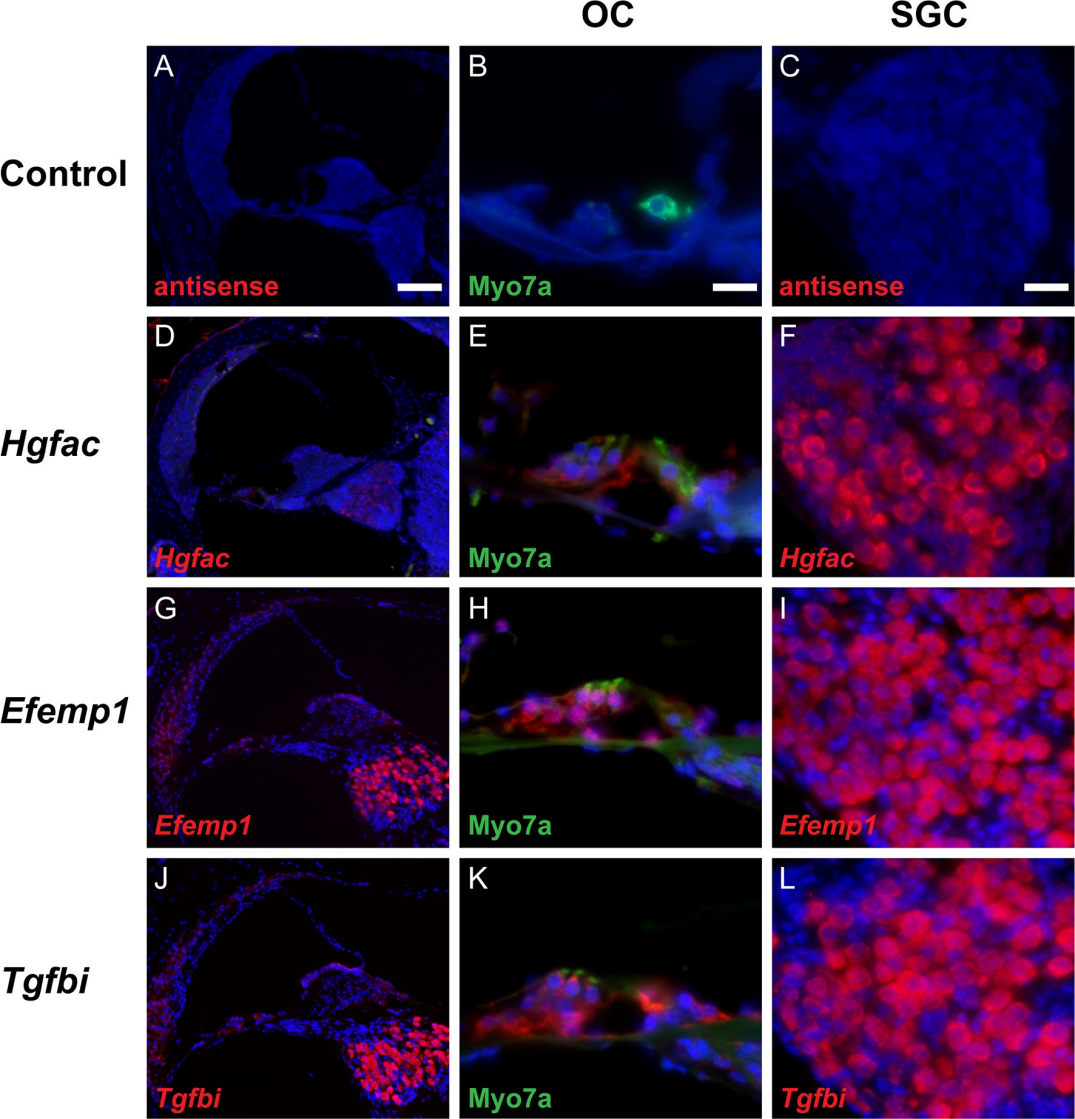
Fluorescence *in situ* hybridization (FISH) of *Hgfac*, *Efemp1*, *Tgfbi* genes in murine cochlear cross sections. (**A-C**) Negative controls for each target gene, hybridized with corresponding antisense probes, revealed no signal. (**D, G, J**) *Hgfac*, *Efemp1* and *Tgfbi* were all localized in the organ of Corti and spiral ganglion, while *Efemp1* and *Tgfbi* were also found in the spiral ligament (**E, H, K**). A magnified view of the organ of Corti revealed that *Hgfac*, *Efemp1*, *and Tgfbi* RNA probes hybridized with IHCs, OHCs and adjacent supporting cells. SGNs were intensely hybridized with *Efemp1* and *Tgfbi* RNA probes (**I, L**), while the signal from *Hgfac* RNA probe was much weaker in the spiral ganglion (**F**). Myosin VIIa was used as a marker of hair cells (green) along with the marker of nuclei, DAPI (blue). Scale bars, 100 μm in the first column; 20 μm in the second and third columns.

Because antibodies targeting HGFAC, EFEMP1 (also known as Fibulin-3) and TGFBI did not produce a reliable signal using immunohistochemistry, we defined tissue localization of the corresponding mRNA transcripts using fluorescence *in situ* hybridization (FISH) applied to murine cochlear cross sections. Control sense RNA probes did not show appreciable hybridization in any part of the cochlea (**Fig 5A–5C** exemplifies this for the *Hgfac* sense probe). Sections hybridized with antisense probes targeting *Hgfac* revealed signals distributed mainly within the organ of Corti and spiral ganglion (**Fig 5D**). A high-magnification view of the organ of Corti revealed strong signal in IHCs, OHCs, and adjacent supporting cells including pillar cells and phalangeal cells (**Fig 5E**). A high-magnification view of the spiral ganglion revealed cytoplasmic expression of *Hgfac* mRNA in neurons with DAPI-stained nuclei (**Fig 5F**).

By contrast, both *Efemp1* and *Tgfbi* probes demonstrated more intense hybridization signals within the spiral ganglion and the spiral ligament compared to that of *Hgfac* (**Fig 5G–5L**). Similar patterns were seen in the organ of Corti: both *Efemp1* and *Tgfbi* probes readily hybridized to inner and outer hair cells as marked by Myosin VIIa, as well as supporting cell populations including Deiter’s cells and pillar cells (**Fig 5H–5K**). There was no observed apical-to-basal gradient in the pattern of RNA probe hybridization for *Efemp1* and *Tgfbi*.

## Discussion

Amongst the existing diagnostic armamentarium for patients with hearing and vestibular disorders, there are few tests that shed light on the underlying molecular pathophysiology. The perilymph fluid represents a promising resource for biomarker discovery and ultimately, the development of new diagnostic and prognostic tools, largely because it is much more highly enriched for molecules secreted by inner ear cells than plasma or CSF. Nevertheless, a systematic comparative analysis between the perilymph proteome in patients with loss of audiovestibular function and those with normal hearing have not been performed. This is largely due to the rare ability to instrument the inner ear with normal hearing, and partly due to inherent technical difficulties in extracting sufficient samples during surgical procedures, as well as limitations in *post-hoc* analysis techniques to quantitatively assess the composition of perilymph.

Our identification of AACT expression in the cochlea is interesting because AACT is a member of the Serpin family of protease inhibitors, and serpins are most abundant proteins in mouse perilymph [19]. Protease inhibitors are major regulators or enzymatic activity and they reduce protease-induced tissue injury. Aberrant protease activation has been associated with inner ear damage and hearing impairment [23]. AACT is one of the most potent inhibitors of leukocyte-derived chymotrypsin-like proteases [24], and it shares approximately 42% sequence homology with α1-antitrypsin [25]. As a circulating plasma glycoprotein encoded by *SERPINA3*, AACT irreversibly binds and thereby inactivates classical chymotrypsin-like target proteases such as neutrophil cathepsin G and mast cell chymase. In multiple models of degenerative diseases including cirrhosis and emphysema, AACT appear to regulate protease-mediated tissue damage and homeostasis [26,27]. Recently, AACT has been identified as a part of the biomarker signature that distinguished between healing and non-healing skin wounds and plays a pivotal role in skin repair [28] [29].

While it is plausible that protease inhibitors play an important role in regulating protease activity in the inner ear, the precise physiological role of AACT in the hearing and balance function has not been reported previously. In a genetically-engineered mouse model carrying mutations in *TMPRSS3*, a serine protease in which mutations are associated with non-syndromic autosomal recessive deafness (DFNB8/10), inactivation of the protease shortly after birth led to severe and rapid degeneration of the organ of Corti and cochlear and saccular hair cells [30]. These results suggest TMPRSS3 may be important for cochlear hair cell survival at the onset of hearing in mice. In situ hybridization studies revealed *Tmprss3* expression in cochlear and vestibular hair cells, supporting cells of the organ of Corti, epithelial cells of the spiral sulcus, and interdental cells [31]. Interestingly in our study, AACT was found to be expressed in similar cell types. Further work is needed to elucidate the mechanism by which AACT regulates protease activity, particularly as it relates to TMPRSS3.

Hepatocyte growth factor (HGF) is important for human hearing because mutations in the genes encoding this protein and its receptor, MET, are associated with hearing loss. Specifically, non-coding mutations of *HGF* are associated with non-syndromic hearing loss, autosomal recessive deafness-39 (DFNB39) [20], while a mutation in *MET* is associated with human DFNB97 hearing loss [32]. Moreover, HGF levels appear to be tightly regulated as too much or too little of it can cause hearing loss [20]. We have therefore focused on validation of HGF activator (HGFAC) expression in the cochlea because HGF is secreted as an inactive precursor and is converted to its active form in response to injury by a family of proteases including HGFAC [33]. HGFAC is known to be generated in liver and circulates as an inactive zymogen, and is activated within the injured tissue which releases activated HGFAC [33]. HGF is a potent factor for cellular regeneration with versatile roles, including mitogenic, angiogenic and morphogenic activities [34,35]. Overexpression of HGF in the spiral ganglion cells in rats *via* viral-mediated intrathecal delivery of human *HGF* gene led to prevention of kanamycin-induced degeneration of hair cells and SGNs [36]. Elsewhere, exogenous HGF had protective effects on auditory hair cells against aminoglycoside-induced ototoxicity, possibly *via* coupling with signaling through the c-MET pathway and by reducing levels of reactive oxygen species [37]. Furthermore, application of HGF directly on the round window protected outer hair cells from noise damage in guinea pigs [35]. Our finding of *Hgfac* expression in cochlear cell types that benefit from exogenous HGF application in animal models provides an insight into the mechanism of this therapeutic effect.

EGF-containing fibulin-like extracellular matrix protein 1 (EFEMP1), also known as Fibulin-3 (FBLN-3), belongs to a family of six proteins associated with the extracellular matrix elastic fibers and basement membranes [38]. We focused on validation of EFEMP1 expression in the cochlea because a gene encoding an effector of EGF-mediated cell signaling causes progressive hearing loss [21], and single mutations in the *EFEMP1* gene cause two inherited forms of macular degeneration [39]. EFEMP1 is widely expressed in tissue rich in elastic fibers, such as the cardiovascular system and small capillaries, where it mediates cell-to-cell and cell-to-matrix interactions and provides structural support for the extracellular matrix [40]. Previous experiments have also shown strong interactions between EFEMP1 and TIMP-3, an inhibitor of metalloproteinases, in the setting of tissue injury to potentially reduce proteolysis and remodeling of the extracellular matrix [41]. Our finding of *Efemp1* RNA expression in the spiral ganglion, spiral ligament, and sensory and supporting cells of the organ of Corti overlaps with the cochlear expression pattern of EGF receptor [42,43]. While the definitive role of EFEMP1 in cochlear function remains to be elucidated, the strong association between EFEMP1 and protease activation and regulation suggests that EFEMP1 may antagonize protease activity to prevent cellular injury in the inner ear.

Transforming growth factor beta induced (TGFBI), a structural homolog of periostin, is an extracellular matrix protein induced by TGF-β and normally expressed in fibroblasts, keratinocytes, and muscle cells [44,45]. The precise function of TGFBI remains to be established. We elected to validate TGFBI expression in the cochlea because mutations in TGF-β are associated with human hearing loss [46], while missense mutations in *TGFBI* underlie several types of corneal dystrophies where abnormal deposits of misfolded proteins and amyloid in the corneal stroma lead to visual impairment [47]. TGFBI is secreted into extracellular space and is predicted to bind fibronectin, collagen and integrins to mediate a multitude of physiologic and pathologic processes [48], including cell adhesion and migration [44], wound healing and response to injury [49], inflammation in osteoarthritis [50], tumorigenesis [51,52], and metastasis [53].

Although no prior studies have linked TGFBI expression with the pathophysiology that underlies disorders of hearing or balance, *Tgfb1* expression was reported in the embryonic cochlea in the cells of the inner (fibrocyte) compartment of the periotic mesenchyme, whose differentiation and expansion is subsequently regulated by the canonical Wnt signaling pathway [54]. Our localization of *Tgfbi* in specific cell types of the adult cochlea validates TGFBI detection in adult human perilymph, and motivates future studies to decipher the role of this protein in cochlear function.

A comprehensive network and pathway analysis of the proteome of normal human perilymph highlighted several pathways of interest. The core proteome consisted of largely immunoglobulins, proteases, and protease inhibitors, suggesting that the perilymph is a tightly-controlled, well-balanced system in regulating inner ear function. The acute phase response is a rapid, protective, inflammatory mechanism triggered by a variety of factors. Regulation of inner ear inflammation is likely a critical function of perilymph proteins, as increased inflammatory cytokine production is associated with SNHL in meningitis, otitis media and bone diseases characterized by pathologic remodeling of the otic capsule, including otosclerosis and Paget’s disease [55,56]. The Nuclear transcription factor κB (NFκB) has fundamental roles in the regulation of this response in many types of cells, including the degeneration of SGNs as well as hearing loss in mice. Animals lacking the p50 subunit of NFκB demonstrated accelerated loss of SGNs and age-related hearing loss[57]. Our finding of retinoic acid signaling as the second top-ranking pathway in normal human perilymph further highlights the importance of this pathway, which was previously recognized in metabolomic analysis of metabolites reported to alleviate SNHL [58]. Retinoid acid levels are likely tightly controlled in the inner ear as either excess or deficiency in retinoid acid can lead to dysmorphogenesis of the inner ear [59].

Analysis of the differentially expressed genes in perilymph from patients with normal hearing compared to those with VD yielded two significant networks of interest: one involved in tissue injury and inflammation, and the other associated with energy production. These networks, along with the relative overabundance of enzymes in VD perilymph compared to normal perilymph, may reflect primary etiology or secondary damage to inner ear cells resulting in release of intracellular contents into perilymph. Several bodies of evidence in the literature support the link between inflammation, cellular degeneration and the pathogenesis of MD. Arenberg *et al*. theorized that inflammation associated with viral infection could affect the stria vascularis, dark cells, and endolymphatic sac in Meniere’s patients [60]. Furthermore, the etiology of Meniere’s disease has also been linked to otitis media where inflammatory products and toxins could infiltrate the perilymphatic space [61]. Additionally, there is a significant increase in the level of circulating immune complexes in Meniere’s patients, which may lead to increased vascular permeability in the stria vascularis or endolymphatic sac [62]. Finally, degenerative and hypoplastic changes in the endolympahtic sac of patients with Meniere’s disease have been recently linked to mineralocorticoid-controlled sodium transport in the inner ear [63], and mineralocorticoid receptor activation is known to play an important role in inflammation [64]. The dark cells in the cristae ampullaris of semicircular canals, cells in the stria vascularis and select cells of the endolymphatic sac harbor high levels of mitochondria and are associated with regulation of electrolyte transport within endolymph, inner ear energy production and energy homeostasis [65]. When temporal bone histopathology between patients with or without labyrinthitis are compared, there is a significant decrease in the number of dark cells in the lateral and posterior semicircular canals in patients with vestibular dysfunction [66]. Furthermore, there is abnormal appearance and decreased density of dark cells and evidence of stria vascularis atrophy in the temporal bones of Meniere’s patients when compared to those with non-Meniere’s hydrops or otherwise normal controls [67–69]. With recent evidence supporting the role of aquaporins [70], and specifically aquaporins 2, 4 and 5 in the pathogenesis of MD, it is relevant that aquaporin 5 is regulated by cyclic adenosine monophosphate (cAMP); other derivatives of adenosine, including adenosine triphosphate (ATP) play important roles in energy transfer. Together, these findings suggest putative roles of mitochondrial-rich cells of the inner ear in contributing to the proteomic landscape of perilymph in patients with vestibular dysfunction.

Our patients with skull base meningiomas had normal or near-normal hearing. Unlike vestibular schwannomas where hearing is often impaired due to a combination of mechanical nerve compression and tumor-secreted factors, and hearing does not typically improve after tumor removal, hearing improvements have been observed after removal of intracananicular meningiomas. A recent histopathological study on human temporal bones with meningiomas of the IAC showed that cochlear damage is uncommon and hair cell loss is rarely detected [71]. Therefore, it is not surprising that the perilymph proteomic profile of patients with IAC meningiomas clustered closely with each other.

Recently, Shew *et al*. reported the microRNA (miRNA) profile of human perilymph isolated from patients undergoing cochlear implantation, stapedectomy, or labyrinthectomy [72]. A total of 108 miRNAs, corresponding to 405 unique genes, were identified across all three procedures. However, the heterogeneity of the disease processes may directly impact the expression patterns of the miRNAs within the perilymph. Further, a proteomic map of normal perilymph in patients with normal hearing has not yet been accomplished. By contrast, our analysis represents the largest comparative study to date of normal human perilymph proteome, as well as the systematic identification of candidate proteins differentially expressed in the perilymph of patients with disabling peripheral audiovestibular dysfunction relative to normal controls.

## Conclusion

In this report, we have assembled the first proteome of normal human perilymph, collected from people with intact hearing, and performed comparative proteomic analysis of perilymph from patients undergoing labyrinthectomies for disabling vestibular dysfunction due to MD with drop attacks. Using tandem LC-MS/MS, we have significantly expanded the landscape of the known perilymph proteome to over 200 proteins, more than doubling candidate proteins found in previous efforts. We further validated 4 novel protein candidates that had not been previously described in the adult inner ear. We analyzed and defined their precise spatial distribution in the adult murine cochlea by immunohistochemistry (AACT) or fluorescent in-situ hybridization (*Efemp1*, *Hgfac*, *Tgfbi*). Findings from this work will motivate the future development of diagnostic and prognostic assays for hearing loss and vestibular dysfunction based on proteomic markers from inner ear fluids.

## Supporting information

S1 Fig Ingenuity pathway analysis (IPA) of perilymph proteins from patients with intact hearing. The top 3 networks (**A-C**) are shown. **A.** Acute phase response signaling canonical pathway, **B.** Liver X receptor (LXR) / retinoid X receptors (RXR) Activation pathway, **C.** Farnesoid X receptor (FXR) / retinoid X receptors (RXR) Activation pathway. There is an overlap of approximately 21.6% - 26.4% of proteins in the pathway with that in the perilymph samples.
(TIFF)

S2 Fig Ingenuity pathway analysis (IPA) of differentially expressed perilymph proteins from patients with intact hearing versus those with vestibular dysfunction. The top 3 networks are shown (**A-C**). **A.** Organismal Injury and Abnormalities, Respiratory Disease, Inflammatory Disease. **B.** Energy Production, Small Molecule Biochemistry, Developmental Disorder. **C.** Cell-To-Cell Signaling and Interaction, Cellular Assembly and Organization, Nervous System Development and Function. ERK 1/2, HSP, and APP were nodal molecules in each of the three networks, respectively.
(TIFF)

S3 Fig Audiometric information from both the ipsilateral (affected) ear and the contralateral ear on patients who participated in the study. PTA, pure tone average in decibels (dB). WR, Word recognition (%).
(TIFF)

S4 Fig Relative spectral counts of AACT, EFEMP1, HGFAC and TGFBI. Dots represent individual samples.
(TIFF)

S1 Table List of 228 identified core perilymph proteins and 159 unique genes from IPA. (XLSX)

S2 Table List of 51 proteins previously identified from pathologic ears and 90 unique proteins added to the database. (XLSX)

S3 Table List of 726 genes from Max-Planck Unified Proteome Database (MAPU) and 88 proteins with overlap. (XLSX)

S4 Table List of 56 genes from mouse perilymph and CSF and 15 proteins with overlap. (XLSX)

S5 Table List of 38 proteins summarized in the Venn diagram that are differentially expressed between perilymph of patients with normal hearing and those with seere vestibular dysfunction. (XLSX)

S6 Table Spectral counts of all perilymph proteins. (XLSX)

S7 Table Spectral counts of four candidate protein markers in normal perilymph and perilymph from patients with severe vestibular dysfunction. (XLSX)

## Abbreviations

AACT: alpha-1 antichymotrypsin
ABR: auditory brainstem responses
CI: cochlear implantation
CSF: cerebrospinal fluid
CT: computed tomography
EFEMP1: EGF-containing fibulin-like extracellular matrix protein 1
FISH: fluorescence in-situ hybridization
HGFAC: hepatocyte growth factor activator
IAC: internal auditory canal
MD: meniere’s disease
miRNA: microRNA
MRI: magnetic resonance imaging
PTA: pure tone average
SGN: spiral ganglion neuron
SNHL: sensorineural hearing loss
TGFBI: transforming growth factor beta induced
VD: vestibular dysfunction
VS: vestibular schwannoma
WD: word discrimination
