# Supplementary figures and images for: Proteome of normal human perilymph and perilymph from people with disabling vertigo
Source: PLoS One. 2019 Jun 11;14(6):e0218292. doi: 10.1371/journal.pone.0218292 (PMC6559673; doi:10.1371/journal.pone.0218292)

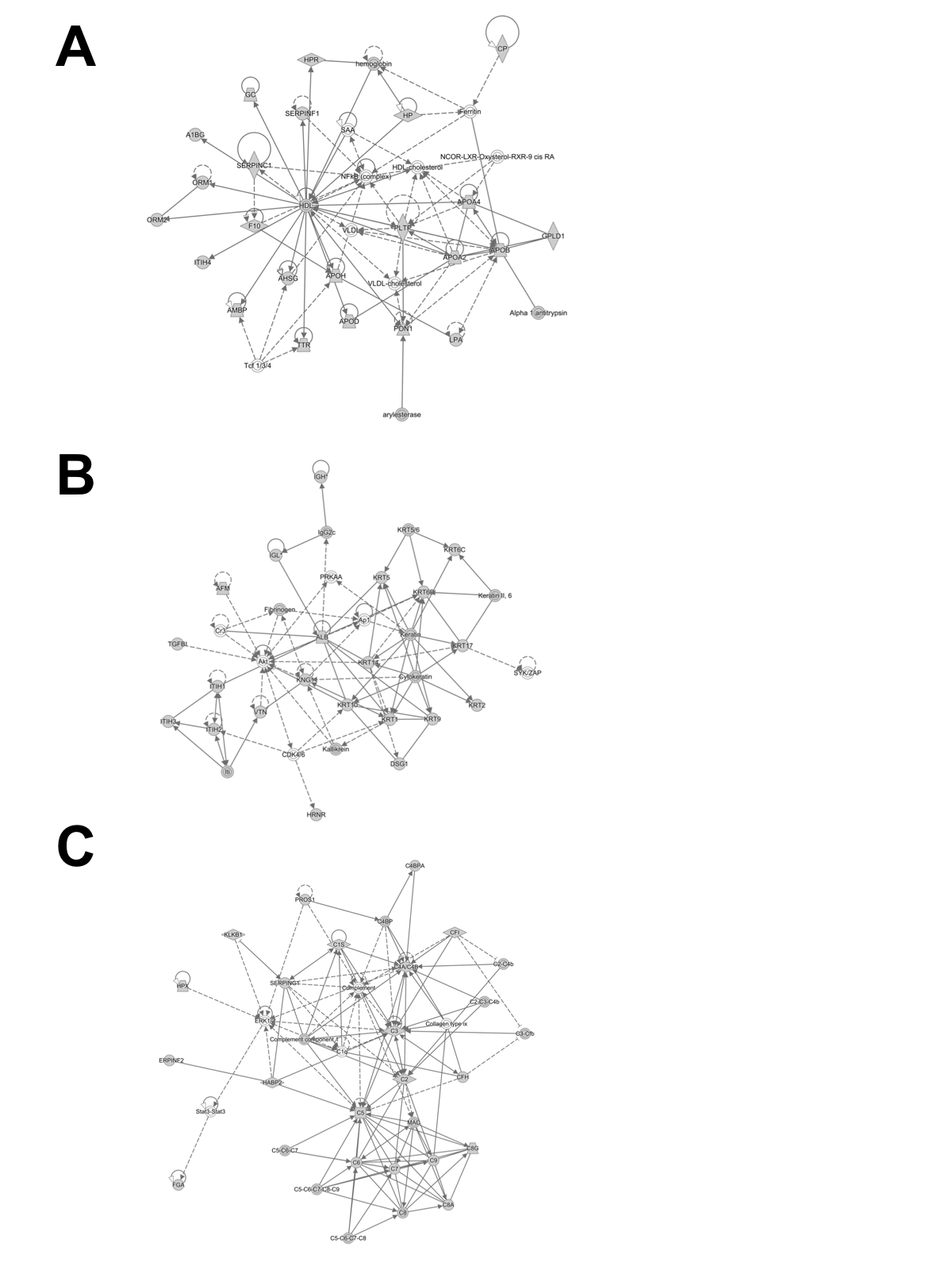

Supplement: S1 Fig — The top 3 networks (A-C) are shown. A. Acute phase response signaling canonical pathway, B. Liver X receptor (LXR) / retinoid X receptors (RXR) Activation pathway, C. Farnesoid X receptor (FXR) / retinoid X receptors (RXR) Activation pathway. There is an overlap of approximately 21.6% - 26.4% of proteins in the pathway with that in the perilymph samples. (TIFF) [file pone.0218292.s001.tiff]

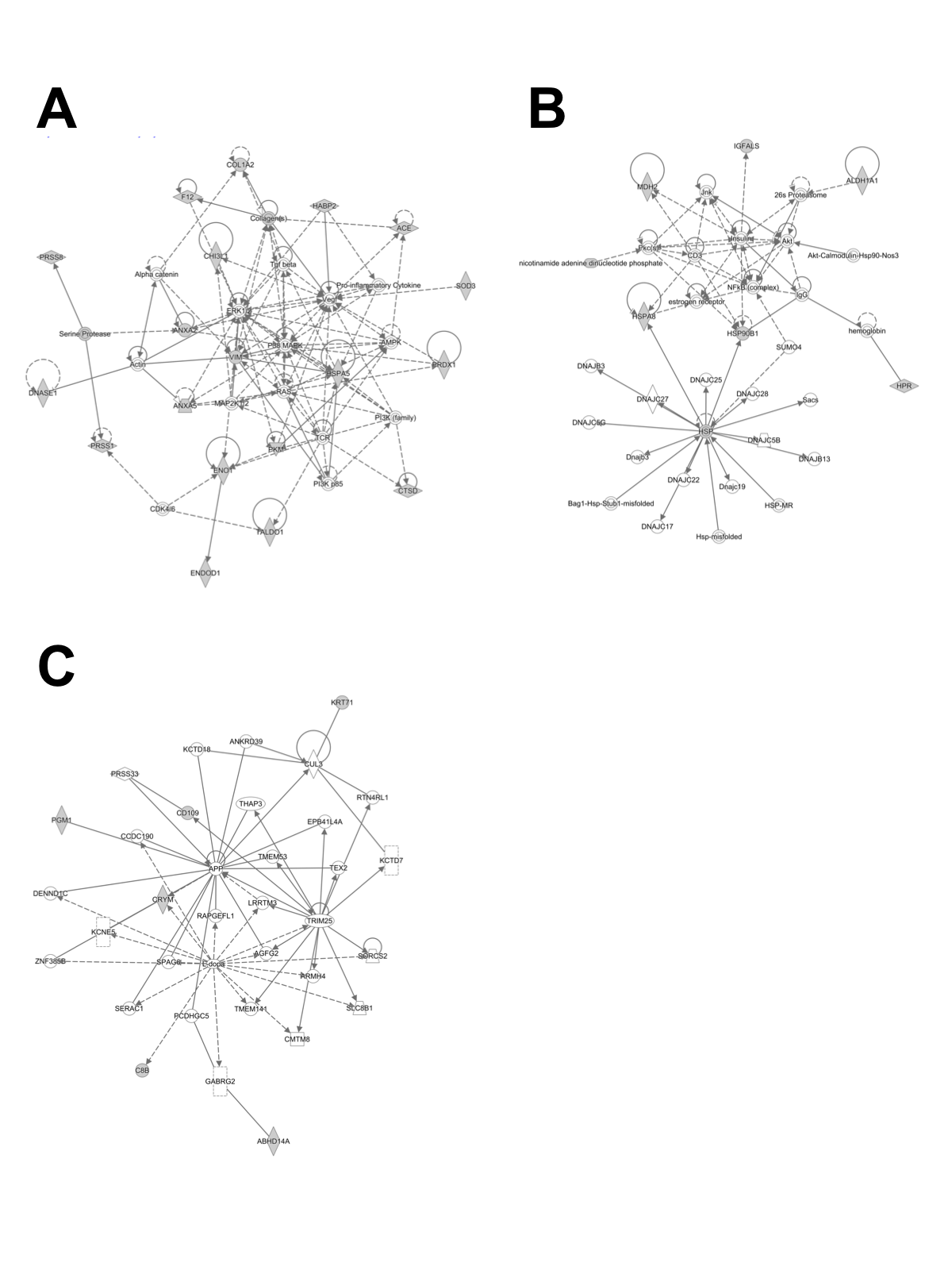

Supplement: S2 Fig — The top 3 networks are shown (A-C). A. Organismal Injury and Abnormalities, Respiratory Disease, Inflammatory Disease. B. Energy Production, Small Molecule Biochemistry, Developmental Disorder. C. Cell-To-Cell Signaling and Interaction, Cellular Assembly and Organization, Nervous System Development and Function. ERK 1/2, HSP, and APP were nodal molecules in each of the three networks, respectively. (TIFF) [file pone.0218292.s002.tiff]

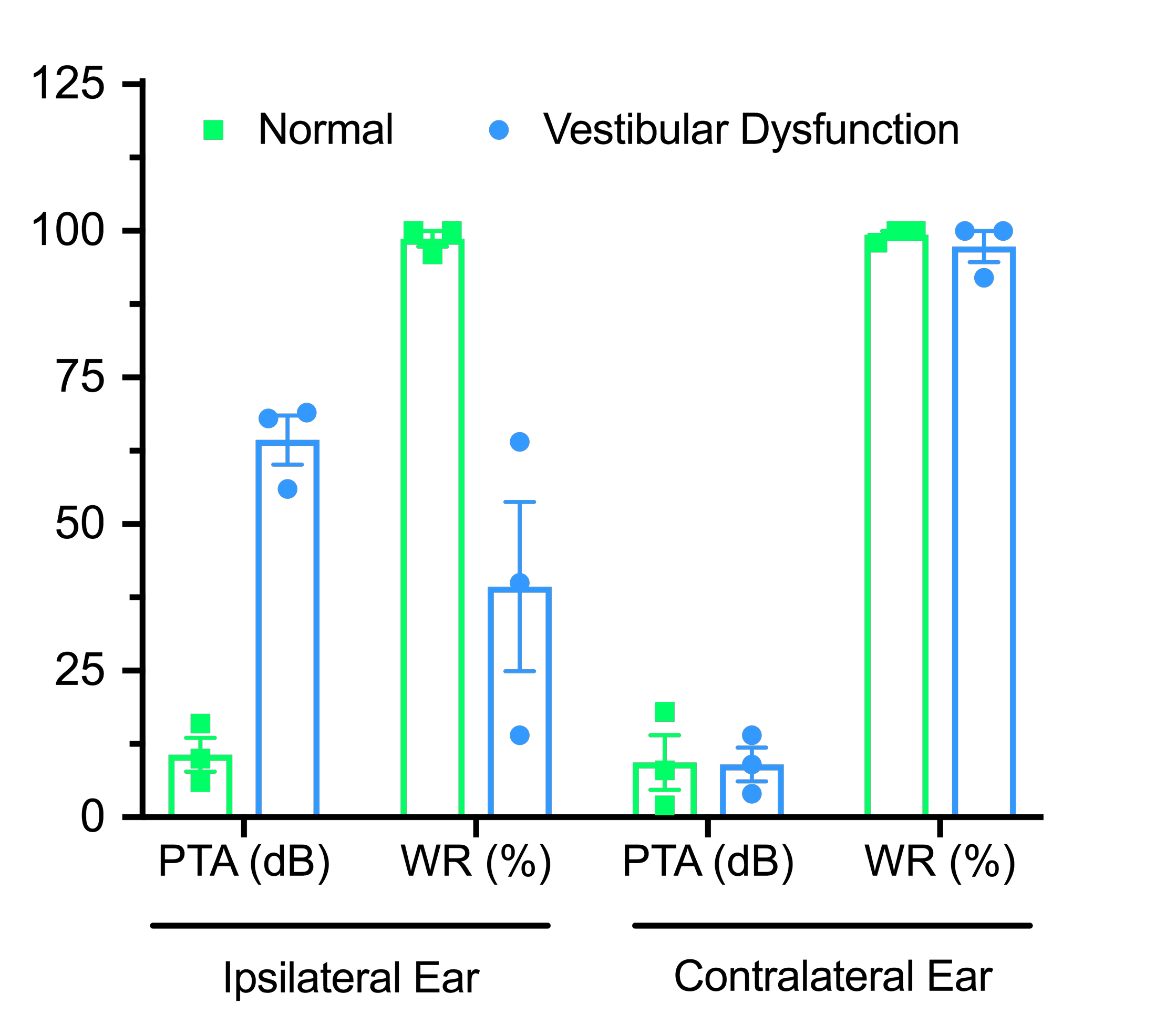

Supplement: S3 Fig — PTA, pure tone average in decibels (dB). WR, Word recognition (%). (TIFF) [file pone.0218292.s003.tiff]

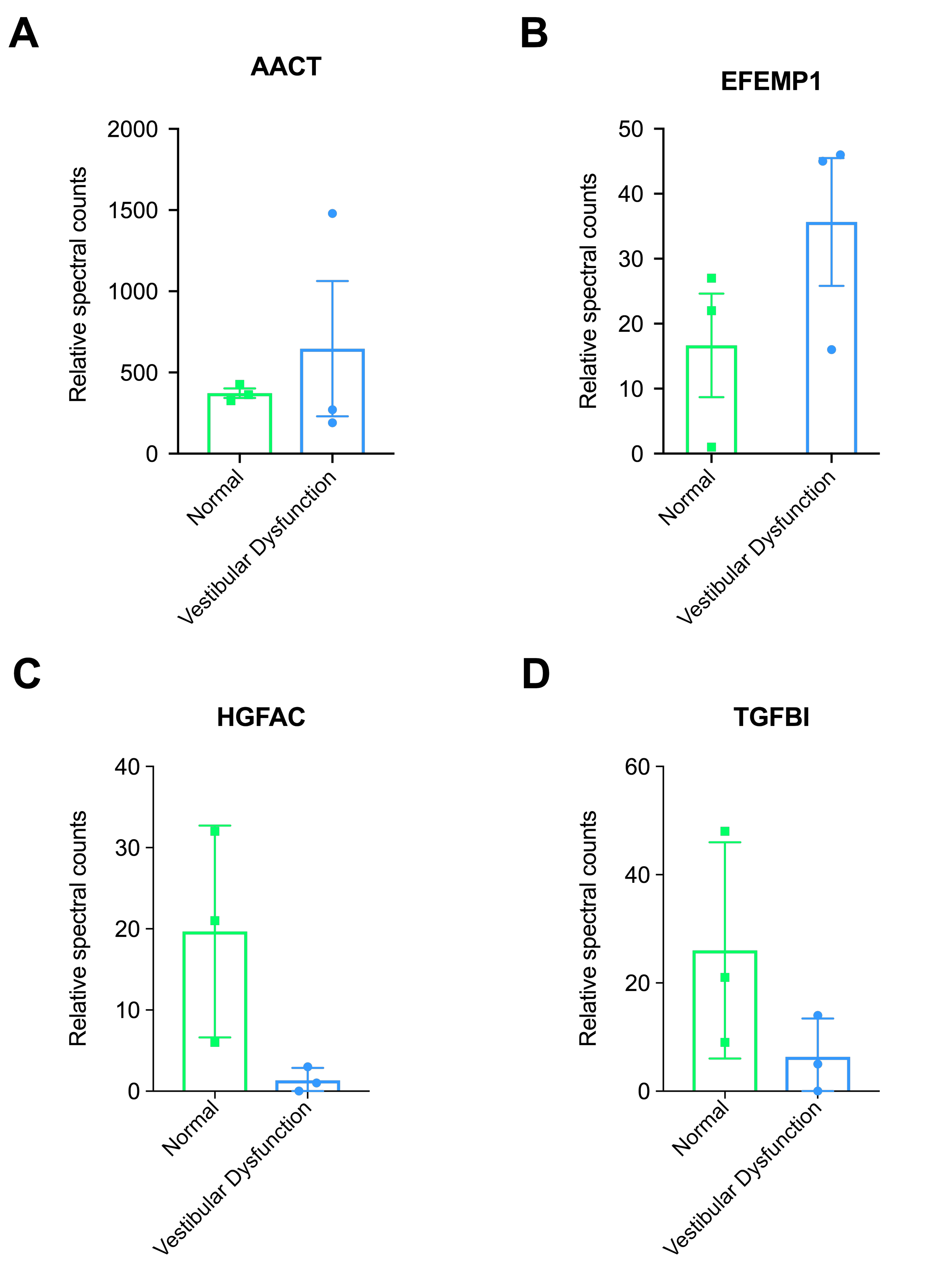

Supplement: S4 Fig — Dots represent individual samples. (TIFF) [file pone.0218292.s004.tiff]
